# An acidic-groups detection method and its application to analysis of Chinese humic acid samples

**DOI:** 10.1371/journal.pone.0238061

**Published:** 2020-08-26

**Authors:** Pengfei Xu, Yuhao Wang, Xue Li, Qingshuang Chen, Lujiang Hao, Jie Zhang, Xiaoling Zhu, Baolei Jia

**Affiliations:** 1 School of Bioengineering, State Key Laboratory of Biobased Material and Green Papermaking, Qilu University of Technology (Shandong Academy of Sciences), Jinan, China; 2 Yinfu (Jinan) Biotechnology Co., Ltd, Jinan, China; 3 Shandong Asia-Pacific Haihua Biotechnology Co., Ltd, Jinan, China; 4 Shandong Academy of Agricultural Sciences, Jinan, China; Tallinn University of Technology, ESTONIA

## Abstract

The method of non-aqueous conductivity titration (NACT) of organic weak acids was applied to quickly and accurately determine the phenolic-hydroxyl and carboxyl-groups contents in humic acid. By varying the pH of the humic-acid sample, the concentration of the titrant, and the nitrogen-gas flow rate, the optimal titration conditions were determined to be a sample pH of 4, titrant concentration of 0.05 mol/L, and nitrogen-gas flow rate of 80 mL/min. Applying the detection method to p-hydroxybenzoic acid showed that its phenolic-hydroxyl content was 758.82±111.76 cmol/kg and carboxyl content was 744.44±51.11 cmol/kg. The theoretical phenolic-hydroxyl and carboxyl-groups contents of the p-hydroxybenzoic acid were 723.96 cmol/kg respectively, indicating that the method can accurately quantify the carboxyl and phenolic-hydroxyl groups in the sample. The NACT was used to measure the phenolic-hydroxyl and carboxyl-groups contents in humic acid quickly and accurately. In addition, 29 humic acid samples from 8 provinces of China covering the main humic-acid producing areas were collected and analyzed for acidic-groups content using the reported method.

## Introduction

**Fig 1:**
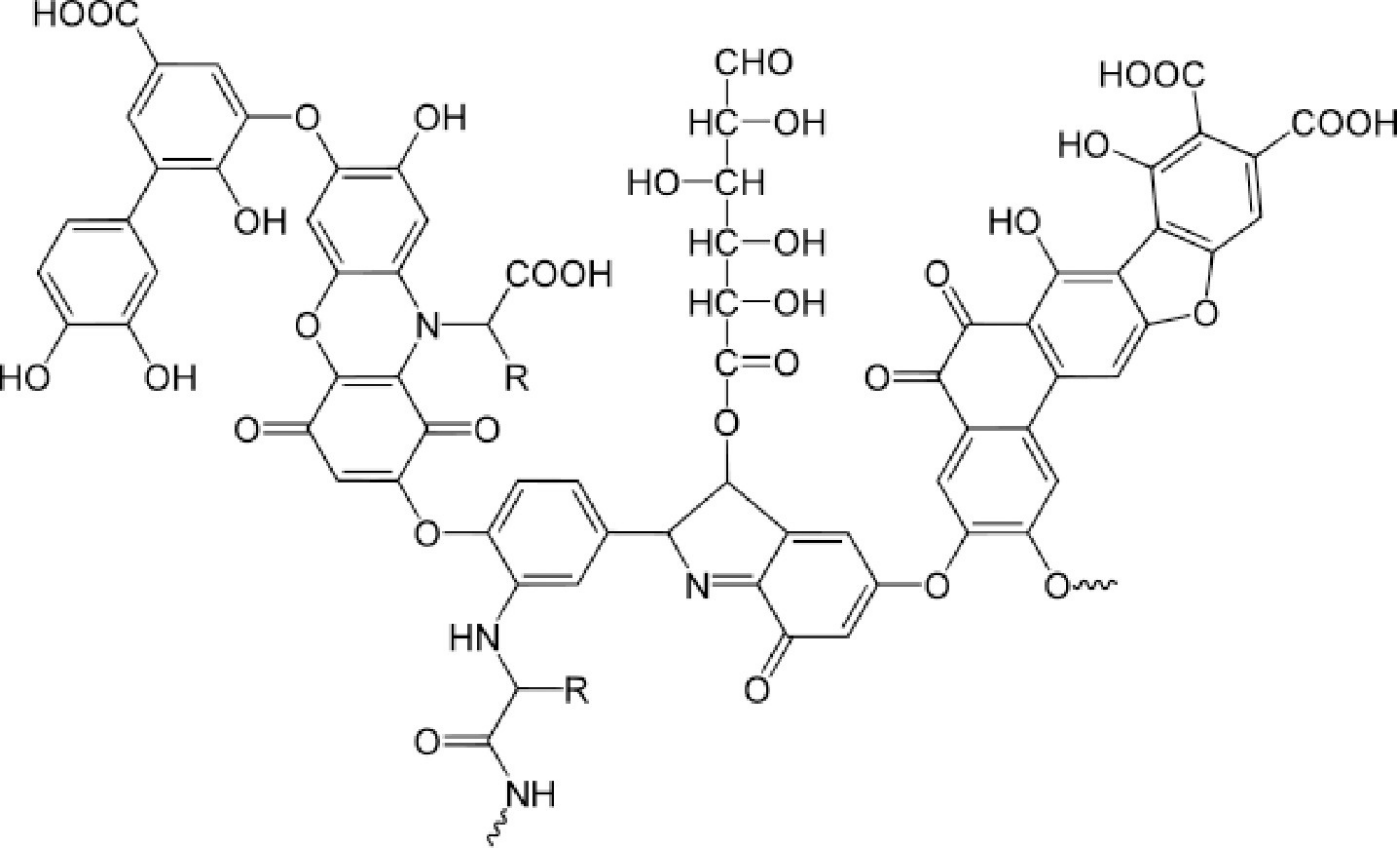
Humic acid model suggested by F. J. Stevenson [13].

Humic substances are complex and heterogeneous mixtures of polydispersed materials formed in coal, soils, sediments, and natural waters by biochemical and chemical reactions during the decay and transformation of plant and microbial remains (a process called humification) [1]. Humic acid, together with fulvic acid and humins are the major component of humic substances. As the acid-insoluble fractions of humic substances, Humic acid is partially soluble in water and form micelle-like structures in neutral to acidic conditions [2]. Humic acid is polymer compound rich in active groups such as phenolic-hydroxyl, carboxyl, sulfhydryl, and carbonyl [3, 4] and is widely used in agriculture, ceramics, oil extraction, and environmental protection as an organic fertilizer, binder, mining aid, water purifier, and heavy-metal remover [5–11]. Humic acid is mainly found naturally in peat, lignite, and weathered coal in nature [12, 13], which composition can vary according to geographical origin, age, climate and biological conditions, making the precise characterization of these substances difficult [14, 15]. Humic acid is highly acidic due principally to carboxyl and phenolic-hydroxyl groups. Total acidity of humic acid may range from 1 mol/kg to more than 14 mol/kg. Other functional groups in humic acid include quinone and ketonic carbonyl, amino, and sulfhydryl groups (Fig 1) [13, 16–23]. These groups are responsible for exhibiting good hemostasis, and antidiarrheal functions. Humic acid is widely used in medicine and feed additives [24–26], and the phenolic-hydroxyl and carboxyl-groups contents in humic acid is positively correlated with its biological activity [27, 28].

By hydrolyzing the phenolic-hydroxyl and carboxyl esters in humic acid, the free phenolic-hydroxyl and carboxyl-groups contents can be increased and the biological activity of the humic acid can be improved, so quantitative analysis of these groups in humic acid is key to its quality control. Folin phenol method and calcium acetate method (FPCA), potentiometric titration, NMR, and infrared method are commonly used to study the acidic groups in humic acid [29–36], but some of these methods are complicated, while some have high detection limits and inaccurate measurement results. For example, the FPCA method can be used to determine the concentrations of phenolic-hydroxyl and carboxyl-groups through classical titrations in the laboratory, however, the two different groups cannot be simultaneously determined, resulting in time-consuming work. Furthermore, NMR spectroscopy and infrared method involves the use of complicated equipment and have limiting operation-technology requirements, making it inconvenient for common application in the production and quality control of humic-acid products.

The non-aqueous conductivity titration (NACT) is the most common titrimetric procedure used in pharmacopoeial assays and it is suitable for the titration of very weak acids and very weak bases, which has been used to quantitatively analyze phenolic-hydroxyl and carboxyl groups in lignin [37–39]. Because humic acid and lignin have similar molecular structures and NACT can be applied to the measurement of weak organic acids [38], in this study, we attempted to adopt this method to measure the phenolic-hydroxyl and carboxyl-groups contents of humic acid by optimizing the humic-acid sample pH, concentration of the titrant, and nitrogen-gas flow rate. It was found that it can be used to simultaneously measure the phenolic-hydroxyl and carboxyl-groups contents in humic acid by one titration, which is convenient and quick.

## Materials and methods

### Test materials

**Fig 2:**
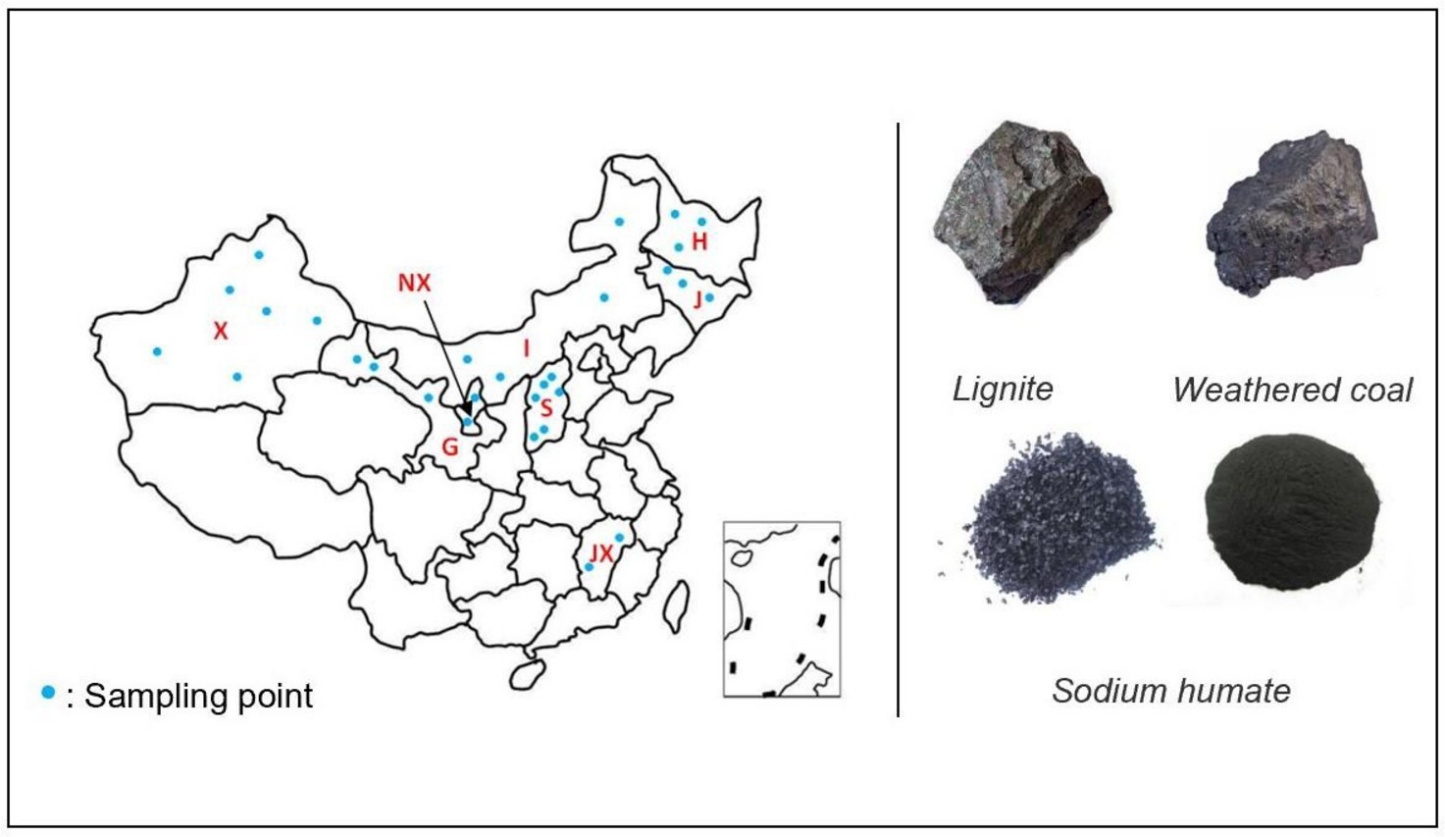
Humic acid products from different producing areas in China. H: Heilongjiang, J: Jilin, I: Inner Mongolia, S: Shanxi, G: Gansu, X: Xinjiang, JX: Jiangxi, and NX: Ningxia.

Samples were collected from the humic-acid producing area of China, as shown in Table 2 and Fig 2. Qingshuang Chen from Shandong Asia-Pacific Haihua Biotechnology Co., Ltd. and Xue Li from Yinfu (Jinan) Biotechnology Co., Ltd. collected, registered, and numbered the humic acid samples from the two companies. The two companies do not make any requirements on the use of humic acid raw materials.

Humic-acid raw materials contain various trace elements and impurities, which would affect the detection accuracy; hence, they are converted into sodium humate by a reaction with sodium hydroxide during the extraction process. Briefly, 50 g humic acid ore powder and 5 g NaOH were mixed in 1000 mL distilled water and stirred at room temperature for 2h. The reaction solution was centrifuged 2800*×g* for 30 minutes to remove the insoluble precipitates. The supernatant was dried at 105°C in oven to a constant weight.

The chemical reagents used in this study included: p-hydroxybenzoic acid, potassium hydroxide, hydrochloric acid, acetone, pyridine, and anhydrous ethanol, which were purchased from Sinopharm Chemical Reagent Co., Ltd. and were all of analytical grade (AR). The main instruments used were: a DDSJ-308F conductivity meter with a lightning magnetic DJS-1C platinum black electrode (Shanghai Lei Magnetic Instrument Factory) and an 85–1 magnetic constant-temperature mixer (Changzhou Guohua Electric Co., Ltd.).

### Preparation of humic-acid sample

10 g of sodium humate was weighed into 100 mL of 12 mol/L hydrochloric acid, stirred well, and then slowly diluted to 300 mL with distilled water. The mixture was then centrifuged at 2800*×g* for 5 minutes.

**Fig 3:**
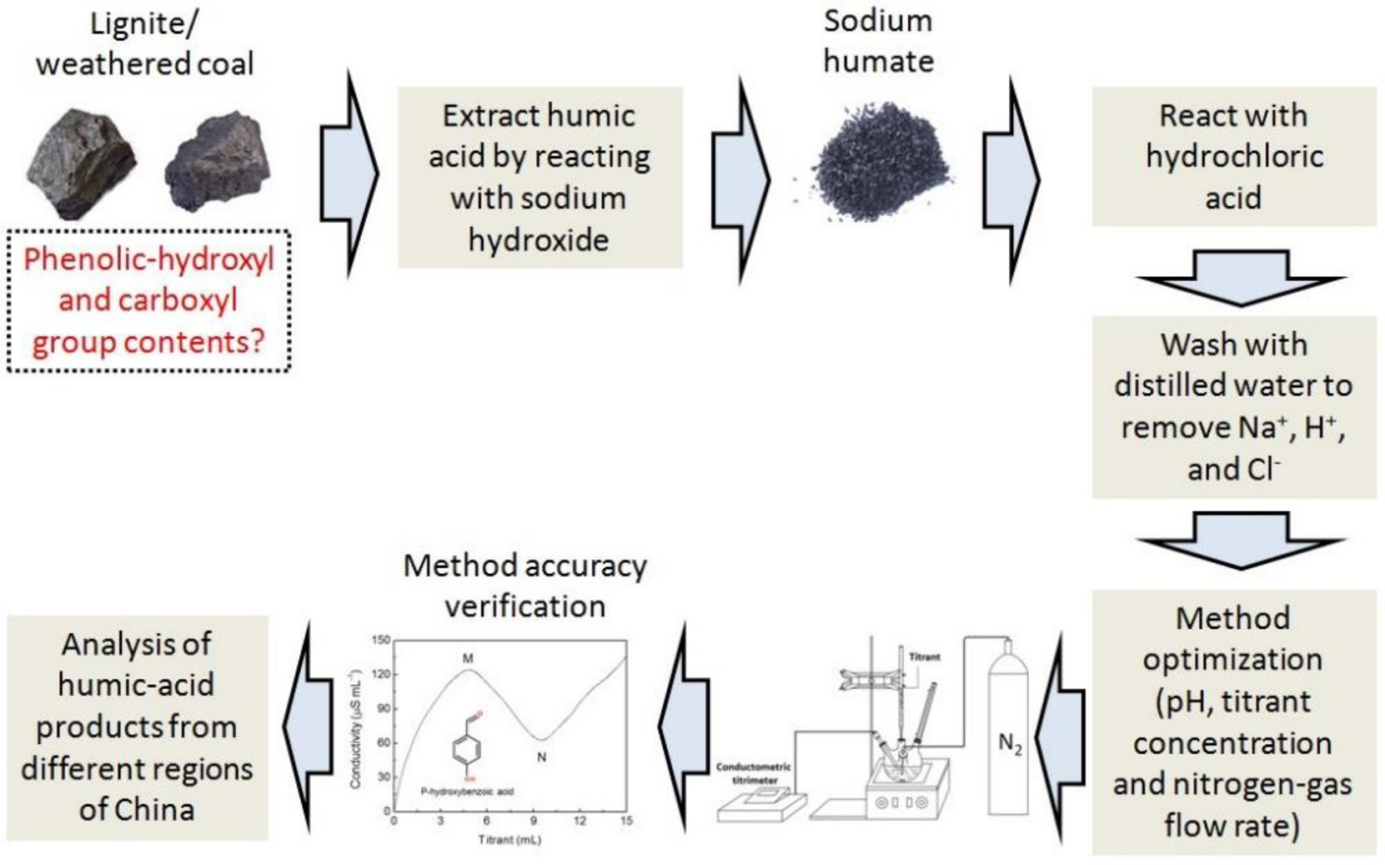
Scheme of the study outline.

A total of 300 mL distilled water was added to the precipitate after centrifugation with thorough stirring. The mixture was centrifuged at 2800*×g* for 5 min, and the pH of the supernatant was measured at the same time. This water-washing process was repeated, followed by drying for concentrate analysis (Fig 3).

### Principle and process of NACT of phenolic-hydroxyl and carboxyl groups in humic acid

During the titration, OH^-^ in the KOH-isopropanol titration solution first reacts with the more acidic carboxyl groups on the humic-acid molecule. After this reaction is complete, the OH^-^ continues to react with the phenolic-hydroxyl groups. The conductivity change of the solution during the reaction is measured using a conductivity meter.

**Fig 4:**
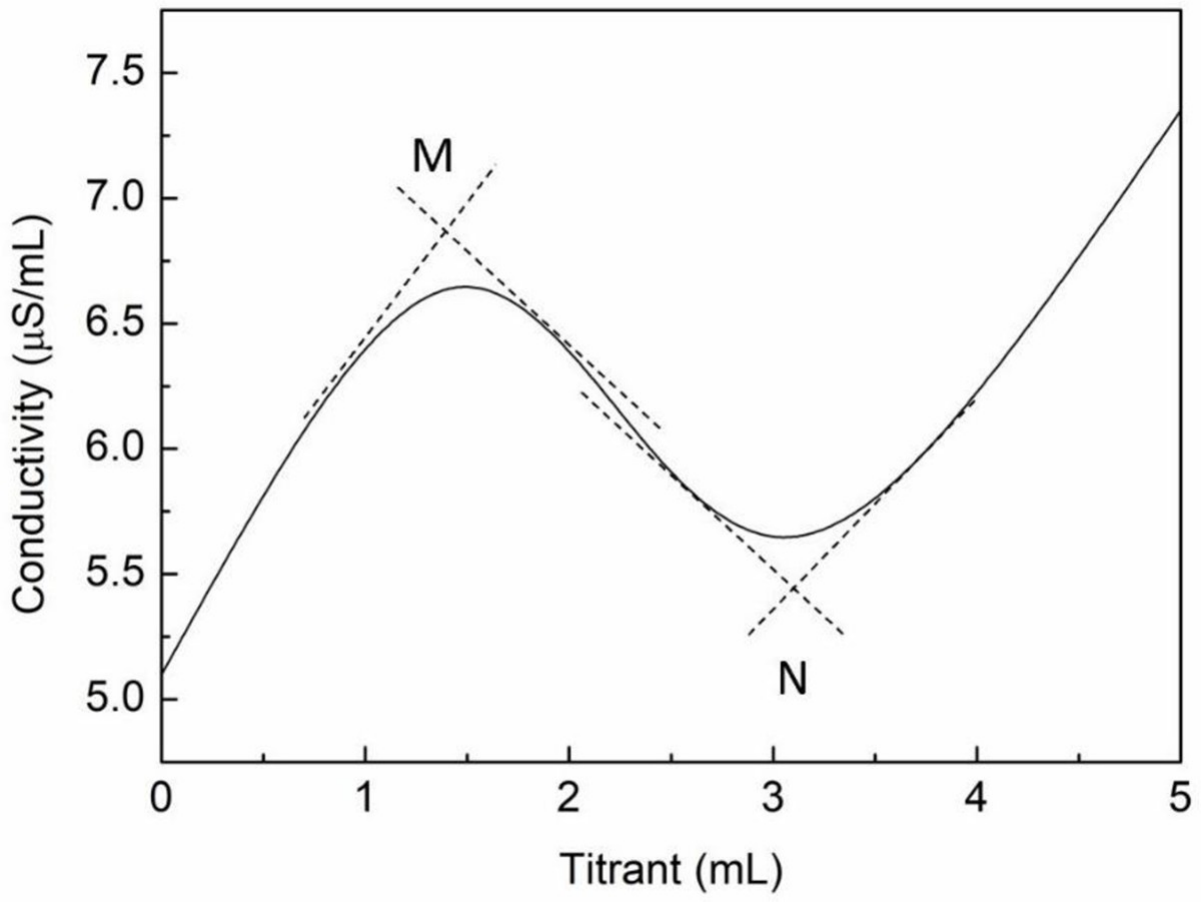
Standard curve for NACT of phenolic-hydroxyl and carboxyl groups in humic acid.

The specific process included weighing 100 mg of humic acid (dry), placing it in a clean and dry 50 mL round-bottom four-necked flask, adding 40 mL of pyridine/acetone (1:4,v:v) solvent and 1 mL of distilled water, and finally adding 0.41 mL ethanol (99.5%, mL/mL). Nitrogen was flowed in to protect the active groups, while the sample was stirred at 200 rpm. After 5 minutes, the titration was performed with a KOH-isopropyl alcohol solution, with the conductivity instrument used to record the conductivity curve until two equivalence points appeared [37]. The equivalence points M and N were determined using the conductivity curve (Fig 4).

The dotted line is the tangent epitaxial line of the inflection point, and the intersection points M and N are the equivalence points of the carboxyl- and phenolic-hydroxyl groups corresponding to the two inflection points, respectively.

Therefore, the carboxyl-groups content A and the phenolic-hydroxyl-groups content P can be calculated using Eqs (1) and (2) on the basis of previous study [39]:

$$A=V_{M}\cdot C\cdot 45/m$$ (1)

$$P=V_{N}\cdot C\cdot 17/m$$ (2)

where *V*_*M*_ and *V*_*N*_ are the titrant consumptions (mL) at the equivalence points M and N, *m* is the sample mass (mg); *C* is the concentration of titrant(mol/L); 45 and17 are the molar masses of the carboxyl group and the phenolic-hydroxyl group, respectively.

### Condition optimization

The sample pH, titrant concentration, and nitrogen-gas flow rate were varied to determine the optimal reaction conditions using the weathered coal from Shanxi province, China (Table 1).

**Table 1:** Condition optimization parameters.

No.	Number of washing cycles (300 mL each)	Titrant concentration (mol/L)	Nitrogen-gas flow rate (mL/min)
1	4	0.03	40
2	6	0.05	60
3	8	0.07	80
4		0.09	100

### Validation of the NACT method

To verify the accuracy of the method for the detection of phenolic-hydroxyl and carboxyl groups, 20 mg p-hydroxybenzoic acid was used as the substrate and the measured phenolic-hydroxyl and carboxyl-groups contents were compared with the theoretical values.

Folin phenol method and calcium acetate method (FPCA) were used to determine the concentration of phenolic hydroxyl groups and carboxyl groups, respectively. In Folin phenol method, 5μL humic acid (2.5 mg/mL) was added into 95μL distilled water. The solution was further mixed with 30μL Folin phenol reagent and 70μL sodium carbonate solution (10%, w/v) in the dark. After reaction at 37°C in the dark for 30 min, the absorbance was measured at 765 nm. p-hydroxybenzoic acid was used as the standard solution to determine the phenolic hydroxyl groups by Folin phenol method. According to the standard curve, the molar number of phenolic hydroxyl in humic acid was calculated, and then the content of phenolic hydroxyl in humic acid sample was obtained. To measure the carboxyl groups, 1g of humic acid sample was added into 130 mL calcium acetate solution (0.25M) in a flat-bottom flask, which reacted for 2 hours at 100 ^o^C in a water bath with condenser tube. Finally, 0.01M sodium hydroxide solution was used to titrated until the pH increase to 8.2. The content of carboxyl groups in humic acid sample was calculated based on that the molar number of sodium hydroxide consumed in titration was equal to that of carboxyl groups in humic acid.

### Statistical analysis

All statistical analyses were performed using SPSS 22.0. A p-value <0.05 was considered a statistically significant result.

## Results and discussion

### Effect of pH and rinsing cycles on conductivity

**Fig 5:**
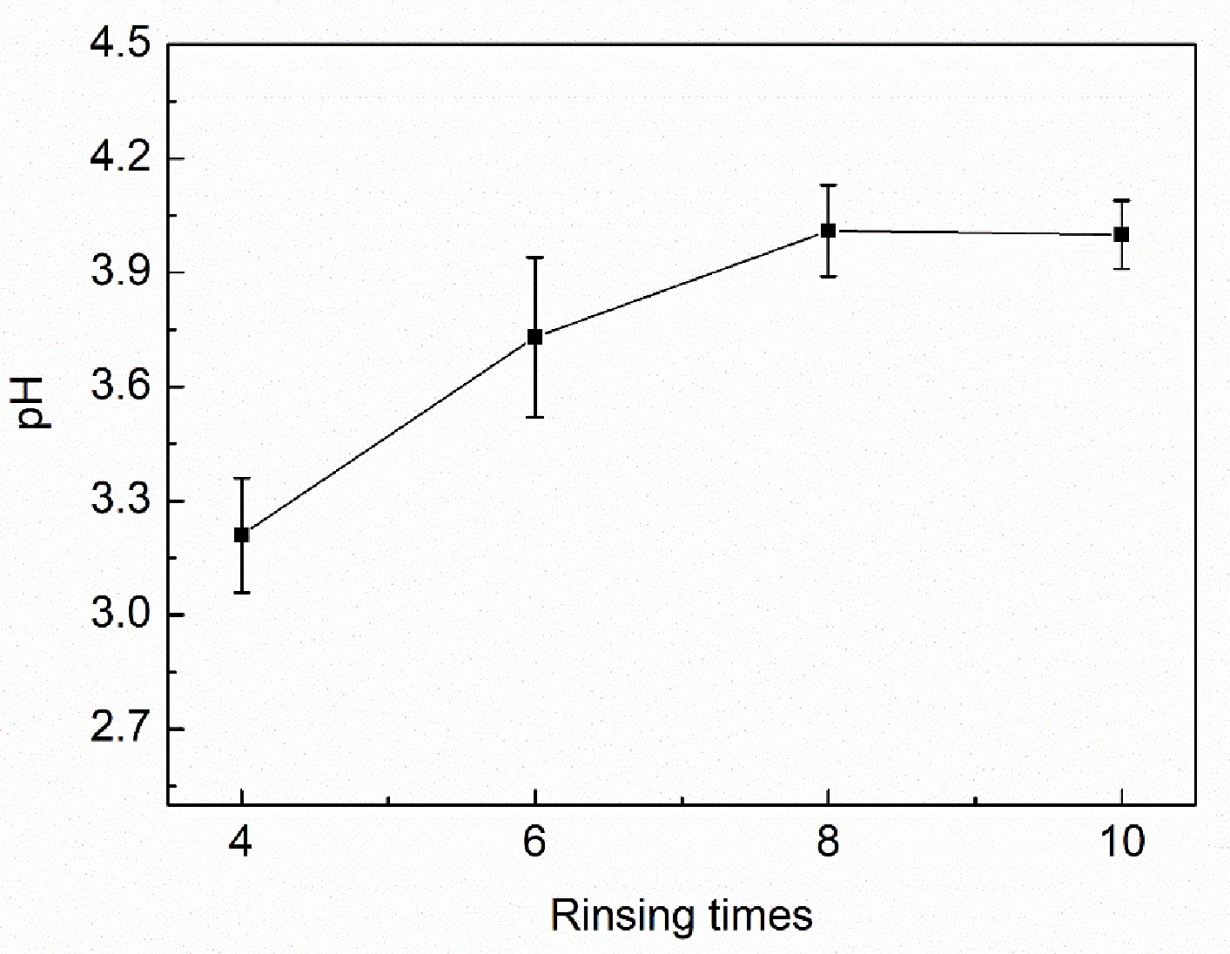
The pH of the humic acid sample after rinsing by distilled water.

The titration cannot be directly performed because the Na^+^ ions in the sodium-humate sample would affect the conductivity of the solution. Instead, insoluble humic acid was formed by reacting the sodium humate with excess hydrochloric acid, and the sample was then washed with distilled water to remove Na^+^, H^+^, Cl^-^, etc., to ensure that no free ions were present in the sample. For convenience, the end point of H^+^ removal was identified by measuring the pH of the supernatant of the washing liquid. It can be seen from Fig 5 that after washing 8 times, the pH of the supernatant no longer changed and the pH of humic acid tended to be stable. This indicated that there was almost no free H^+^ and Cl^-^ remaining in the humic acid sample, so it had reached the requirement for titration. Therefore, in the rest of the study, the samples were washed with 2.4 L of distilled water 8 times to remove free ions.

**Fig 6:**
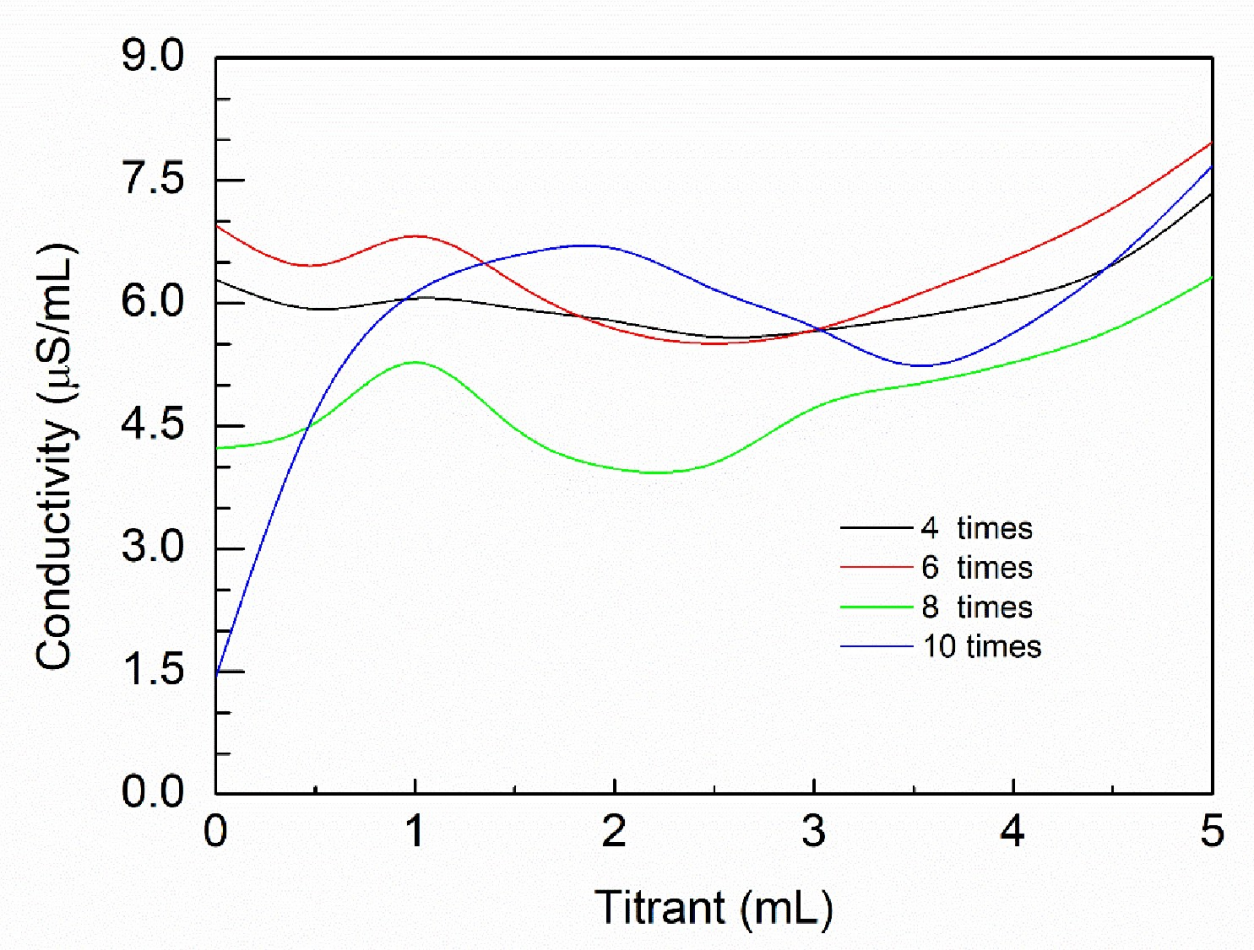
Influence of washing times on conductivity curves.

It can be seen from Fig 6 that the pH value of the sample had a great influence on the conductivity. With 4–6 washing cycles (pH<4.0), there were three inflection points in the conductivity curve, which could be because the residual H^+^ and Cl^-^ affecting the ion concentration in the solution that further affect the conductometric curves. The conductivity curve was more clearly defined after washing 8–10 times (pH≈4) as most of the residuals were removed. However, the humic-acid content in the sample was 55±1.2% after 8 washing cycles, and it was 51±0.9% after 10 washes, indicating that humic-acid sample loss occurred during washing. As long as the pH is stable, the number of washings should be as low as possible to minimize sample loss, so 8 water-washing cycles was optimal.

Therefore, in order to simplify the analysis process, in future experiments, the number of washing cycles and distilled water consumption need not be measured. Instead, the supernatant pH value of 4.0 could be directly used to determine whether H^+^ in the sample had reached the removal end point.

### Effect of titrant concentration on the conductivity curve

**Fig 7:**
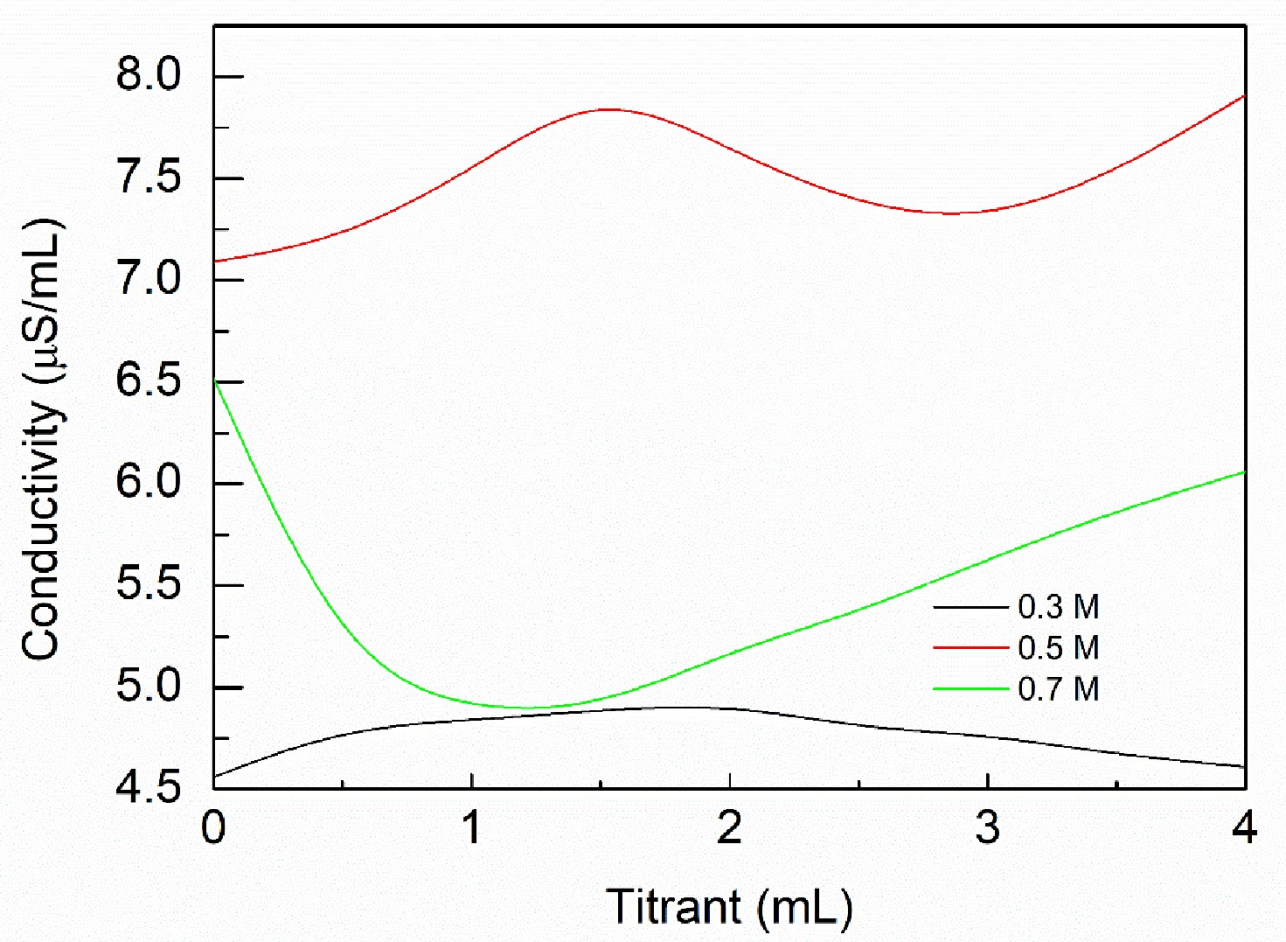
Influence of titrant concentrations on conductivity curve.

The titrant concentration had a strong influence on the conductivity measurements, as shown in Fig 7. When the titrant concentration was 0.03 M, the second equivalence point did not appear. This may be because the concentration of the titration solution was too low and the phenol hydroxyl titration equivalent point cannot be reached. As the volume of the reaction system was limited, we increased the concentration of the titrants. When the titrant concentration was 0.05 M, both the equivalence points appeared. However, when the titrant solution concentration was 0.07 M, the OH-concentration was higher compared to that of the carboxyl content in the sample, so the reaction jumped past the first equivalence point. This made it impossible to calculate the carboxyl content. Therefore, the titrant concentration of 0.05 M was used for the next experimental process.

### Effect of nitrogen-gas flow rate on conductivity

**Fig 8:**
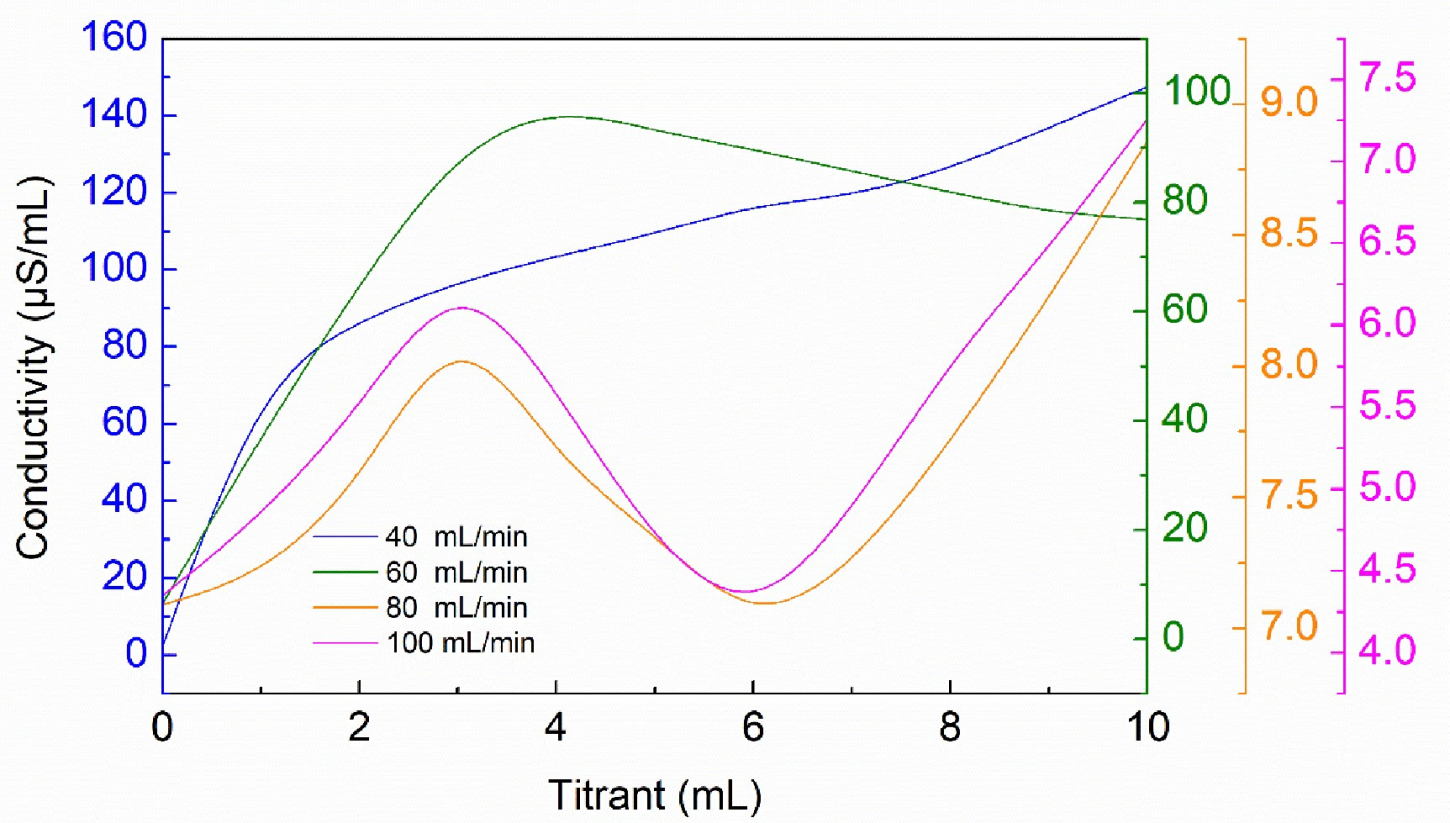
Influence of nitrogen-gas flow rates on conductivity curves.

Phenolic hydroxyl groups in humic acid were easily oxidized to quinone groups during titration with the increase of pH value [40, 41], and the humic acid could be converted to short chain fatty acids such as acetic acid and oxalic acid during the oxidation process [42]. Nitrogen-gas was injected to avoid an undesired oxidation. Fig 8 shows that the nitrogen-gas flow rate had a significant influence on the conductivity curve. The nitrogen-gas flow rate of 40 mL/min and 60mL/min were too low so the reaction did not go to completion or the acidic groups were destroyed by oxidation [41], while the nitrogen-gas flow rate of 40 mL/min and 60mL/min allowed for full reaction of the humic acid. When the nitrogen-gas flow rate was 80 mL/min, the phenolic-hydroxyl-groups and carboxyl-groups contents were measured to be 105.88±17.65 cmol/kg and 60±4.44 cmol/kg respectively, and with the nitrogen flow rate at 100 mL/min, they were 117.65±35.29 cmol/kg and 62.22±8.89 cmol/kg. It can be seen that the measured functional groups contents were very similar. Considering that evaporation of the solvent under a too-high nitrogen-gas flow rate would affect the conductivity, the optimum nitrogen-gas flow rate was identified to be 80 mL/min.

### Verify the accuracy of the method using p-hydroxybenzoic acid

**Fig 9:**
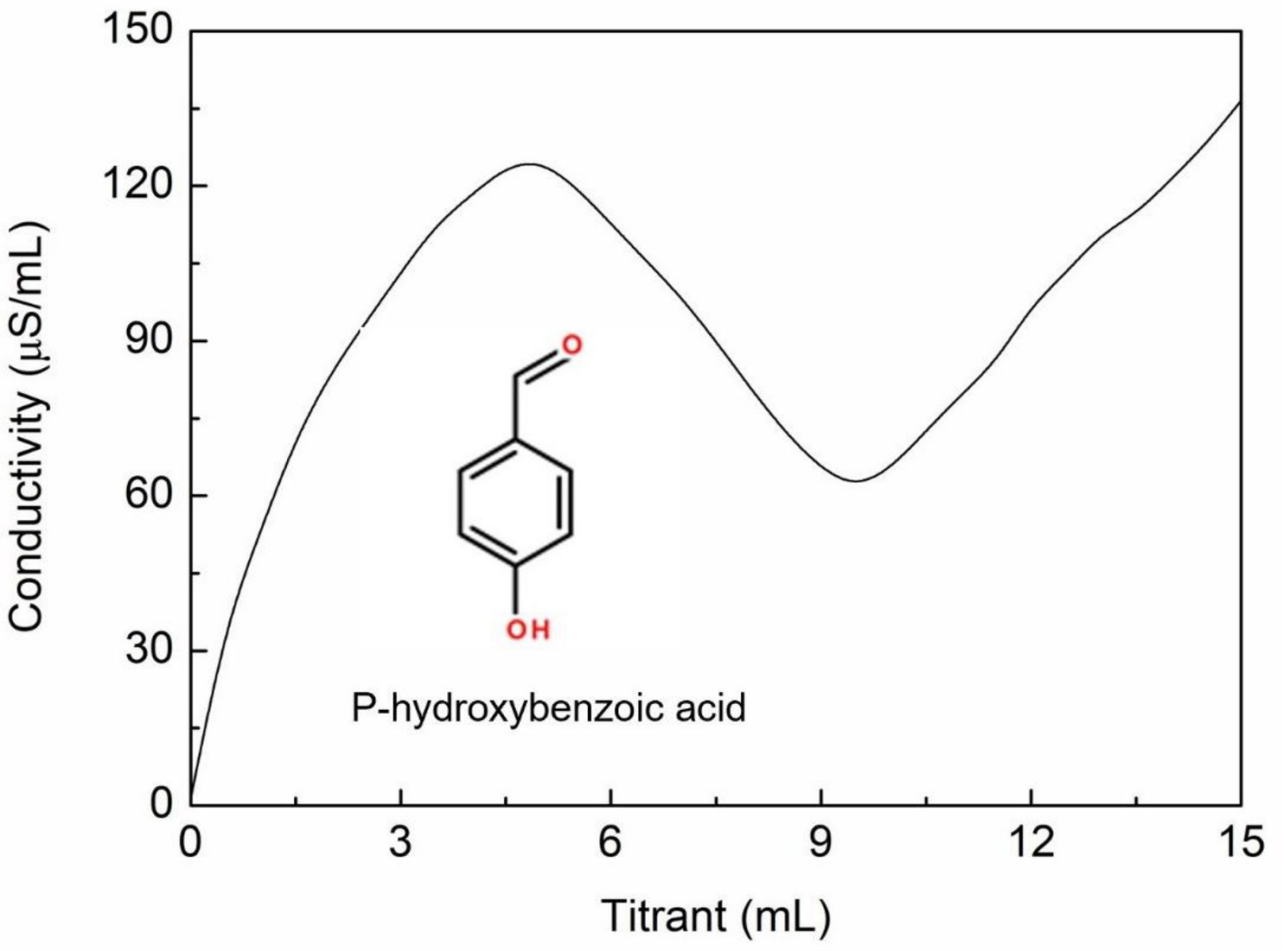
Conductivity curve for p-Hydroxybenzoic acid.

Because the acidic-groups content in humic acid is uncertain, the phenolic-hydroxyl and carboxyl-groups contents of the reference compound p-hydroxybenzoic acid were determined to further verify the accuracy of the NACT. The theoretical carboxyl and phenolic-hydroxyl content in p-hydroxybenzoic acid was 723.96 cmol/kg respectively. The equivalence points M and N of the conductivity curve were determined (Fig 9), wherein M is the carboxyl equivalence point and N is that for phenolic-hydroxyl groups or similar. The carboxyl-groups content A and the phenolic-hydroxyl-groups content P can thus be calculated using Eqs (1) and (2), respectively. In three parallel measurements, the phenolic-hydroxyl and carboxyl-groups contents of p-hydroxybenzoic acid were found to be 758.82±111.76 cmol/kg and 744.44±51.11 cmol/kg. These results indicated that the NACT could be used quickly and accurately to measure the phenolic-hydroxyl and carboxyl-groups contents in p-hydroxybenzoic acid after optimizing the conditions.

### Validation of the NACT method by FPCA method

**Fig 10:**
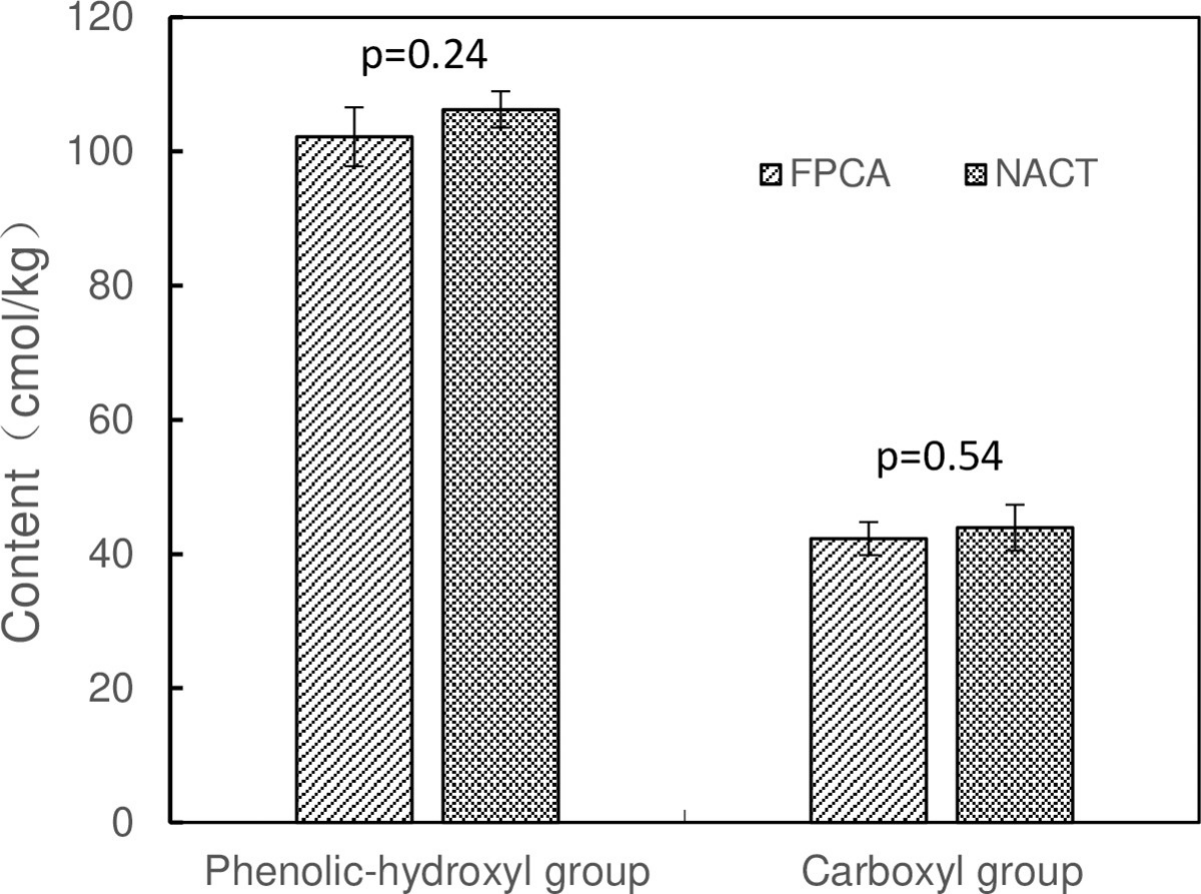
Comparison of NACT and FPCA methods in humic acid analysis.

To validate the data from NACT method, FPCA method was further carried out to measure the phenolic hydroxyl groups and carboxyl groups in the purchased humic acid standard (Fig 10). The result showed that the phenolic hydroxyl groups were 106.26±2.71 and 102.19±4.39 cmol/kg determined by NACT method and by FPCA method, respectively. While the carboxyl groups were 43.93±3.42 and 42.31±2.46 cmol/kg measured by the two methods. The data from the two methods did not show significant difference (phenolic hydroxyl group, p = 0.24; carboxyl group, p = 0.54), which indicated that NACT method is as accurate as previous method.

### Measurement of phenolic-hydroxyl and carboxyl-groups contents in humic acid products from different production areas in China

Quantitative analysis of phenolic-hydroxyl and carboxyl groups in humic acid products from different mining areas in China was carried out using the NACT established in this study, with the results shown in Table 2.

**Table 2:** Analysis of humic acid products from different regions of China.

Source	No.	Content (%)	Content (cmol/kg)	
		Humic acid	Phenolic-hydroxyl group	Carboxyl group
Heilongjiang	H1	25.1±0.53	94.12±25.88	64.44±9.11
	H2	27.7±0.39	88.24±25.29	68.89±10.22
	H3	56.2±1.49	88.24±26.47	42.22±11.33
Jilin	J1	40.4±1.52	129.41±27.65	55.56±11.78
	J2	47.1±0.42	82.35±22.94	28.89±9.78
	J3	56.8±0.36	82.35±17.06	66.67±6.89
Shanxi	S1	42.4±1.21	147.06±6.47	75.56±2.89
	S2	50.5±0.98	111.76±22.35	64.44±8.89
	S3	51.7±0.32	64.71±15.88	57.78±5.56
	S4	59.9±1.73	105.88±24.71	62.22±5.11
	S5	62.9±0.42	111.76±14.12	64.44±4.22
	S6	63.1±0.73	141.18±29.41	68.89±12.44
Xinjiang	X1	52.3±0.97	194.12±11.18	37.78±8.00
	X2	55.9±0.28	105.88±16.47	66.67±6.22
	X3	61.9±0.51	105.88±15.29	60.00±6.67
	X4	65.9±0.43	82.35±12.94	60.00±5.11
	X5	69.4±0.35	123.53±12.94	64.44±5.78
	X6	71.8±0.98	82.35±17.06	68.89±15.11
Inner Mongolia	N1	53.0±0.58	88.24±50.00	53.33±5.78
	N2	55.1±0.50	105.88±22.94	84.44±9.11
	N3	56.7±0.74	152.94±8.24	64.44±7.56
	N4	64.3±0.62	47.59±30.59	51.11±13.33
Gansu	G1	56.5±0.92	82.35±43.53	60.00±10.00
	G2	60.8±1.74	76.47±14.12	57.78±11.78
	G3	63.5±0.87	64.71±22.94	44.44±16.44
Jiangxi	JX1	57.8±0.90	105.88±34.71	75.56±5.33
	JX2	64.3±0.53	94.12±15.29	55.56±5.33
Ningxia	NX1	57.9±0.94	82.35±13.53	66.67±9.56
	NX2	59.6±0.73	88.24±27.06	40.00±4.44

China has a vast territory with large geological differences. According to available data, the distribution of humic-acid mines in China is very uneven, and they are mainly found in Inner Mongolia, Shanxi, Ningxia, Xinjiang, Gansu, Heilongjiang, Jiangxi, and a few other provinces. Humic acid is a complex macromolecular substance that is buried in the ground during the movement of the earth's crust and undergoes a combination of physical, chemical, and microbial processes. Therefore, the structure of the humic acid in each region varies because of the differences in the geological conditions.

**Fig 11:**
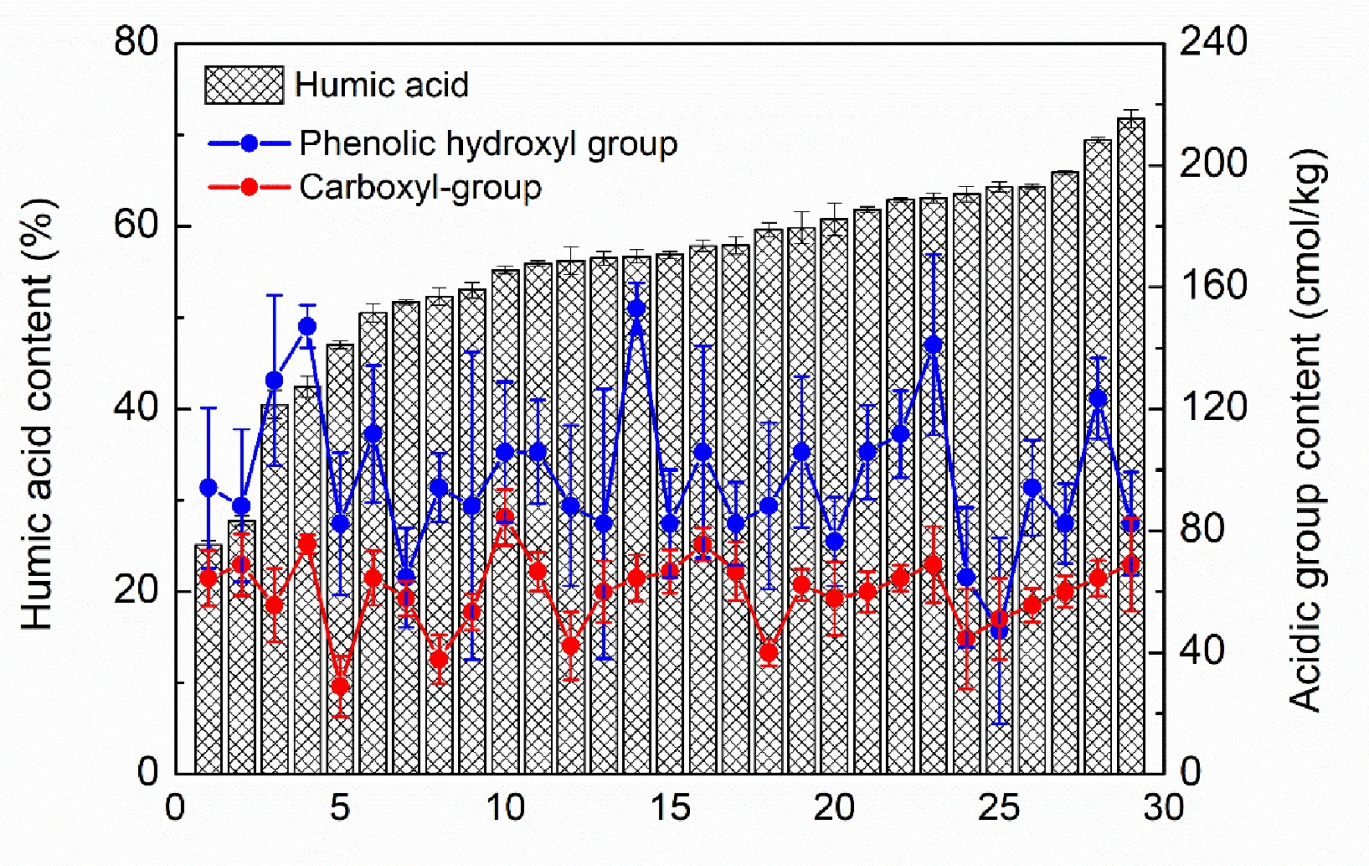
Phenolic-hydroxyl and carboxyl-groups contents in humic acid products.

It can be seen in Table 2 that the humic-acid content in the 29 samples varied from 25.1±0.53% to 71.8±0.98%. There was no obvious correlation between the phenolic-hydroxyl and carboxyl-groups contents and the humic-acid content in the 29 samples. For example, in sample H1 with the lowest humic-acid content, the phenolic-hydroxyl and carboxyl-groups contents were 94.12±25.88 cmol/kg and 64.44±9.11 cmol/kg, respectively, while the humic-acid content was 71.8±0.98 in sample X6, the phenolic-hydroxyl and carboxyl-groups contents were 82.35±17.06 cmol/kg and 68.89±15.11 cmol/kg. It can be seen that there was no obvious correlation between the humic acid and acidic-groups contents in the results (Fig 11).

## Conclusions

For quick and effective determination of the phenolic-hydroxyl and carboxyl-groups contents in humic acid, the measurement of the conductivity curve of humic-acid samples prepared at pH 4 was found to be the most accurate approach. The optimum conditions for the titration process were titrant concentration of 0.05 mol/L and nitrogen gas permeation rate of 80 mL/min.

The NACT was applied to the reference compound p-hydroxybenzoic acid, giving a phenolic-hydroxyl-groups content of 758.82±111.76 cmol/kg and a carboxyl-groups content of 744.44±51.11 cmol/kg, identical to the theoretical phenolic-hydroxyl and carboxyl-groups contents. This method could be used to quickly and accurately measure the phenolic-hydroxyl and carboxyl-groups contents in p-hydroxybenzoic acid, thereby indicating method accuracy and feasibility.

NACT is a new method for measuring the phenolic-hydroxyl and carboxyl-groups contents in humic acid. It is fast, simple, and accurate and facilitates the simultaneous measurement of the phenolic-hydroxyl and carboxyl-groups contents by one titration, which can greatly improve detection efficiency.

The NACT was successfully conducted in this study to determine the acidic-groups content in the samples obtained from the main humic-acid mines in China, demonstrating its applicability for the molecular-structure analysis and functional development of humic acid.

## Supporting information

S1 Data (XLSX)

## References

[1] Felbeck GT. Humic substances in soil, sediment, and water. 1986 12; *Organic Geochemistry*. 9(4), 213 10.1016/0146-6380(86)90071-9

[2] Philippe CB, Michelle W. The (bio)chemistry of soil humus and humic Substances: why is the “new view” still considered novel after more than 80 years? 2019 3; *Front. Environ. Sci*. 10.3389/fenvs.2019.00068

[3] Martina K. Size and charge evaluation of standard humic and fulvic acids as crucial factors to determine their environmental behavior and impact. 2018 7; *Front*. *Chem*. 10.3389/fchem.2018.00235 PMC6041962 30027090

[4] Wang RQ, Gutierrez L, Choon NS, Croué JP. Hydrophilic interaction liquid chromatography method for measuring the composition of aquatic humic substances. 2015 1; *Anal*. *Chim*. *Acta*. 853, 608–616. 10.1016/j.aca.2014.09.026 25467510

[5] Capcarova M, Kalafova A, Lajdova Z, Schwarzova M, Zbynovska K, Hrncar C, et al Effectiveness of non-antibiotic stimulators in Japanese quail diet: Gender comparison and economical annex. 2017 1; *Biologia*. 72(1), 96–104. 10.1515/biolog-2017-0004

[6] Tohid T, Hasan G, Alireza T. Efficacy of mannanoligosaccharides and humate on immune response to Avian Influenza (H9) disease vaccination in broiler chickens. 2010 12; *Vetres*. *Commum*. 34(8), 709–717. 10.1007/s11259-010-9444-8 20872288

[7] Sellamuthu KM, Govindaswamy M. Effect of fertiliser and humic acid on rhizosphere microorganisms and soil enzymes at an early stage of sugarcane growth. 2003 12; *Sugar*. *Tech*. 5(4), 273–277. 10.1007/bf02942484

[8] Arai S, Kumada K. An interpretation of the conductometric titration curve of humic acid. *Geoderma*. 1977 9; 19(1), 21–35. 10.1016/0016-7061(77)90011-8

[9] Li Y, Yue Q, Gao B. Adsorption kinetics and desorption of Cu(II) and Zn(II) from aqueous solution onto humic acid. 2010 6; *J*. *Hazard*. *Mater*. 178(1–3), 455–461. 10.1016/j.jhazmat.2010.01.103 20149528

[10] Jia B, Park D, Hahn Y, Jeon CO. Metagenomic analysis of the human microbiome reveals the association between the abundance of gut bile salt hydrolases and host health. 2020 4; *Gut*. *Microbes*. 11(5), 1–14. 10.1080/19490976.2020.1748261 32329665 PMC7524343

[11] Jia B, Jeon C O. Promotion and induction of liver cancer by gut microbiome-mediated modulation of bile acids. 2019 9; *PLOS Pathog*. 15(9), e1007954 10.1371/journal.ppat.1007954 31487329 PMC6728016

[12] Hayes MHB, Swift RS, Wardle RE, Brown JK. Humic materials from an organic soil: A comparison of extractants and of properties of extracts. 1975 4; *Geoderma*. 13(3), 231–245. 10.1016/0016-7061(75)90020-8

[13] Stevenson FJ. Humus chemistry: Genesis, composition, reactions. 1994 4; 2nd Edition, John Wiley, New York, 95–97. 10.1021/ed072pA93.6

[14] Calmon C. Humic substances in soil, sediment and water. 1986 12; Reactive Polymers, Ion Exchangers, Sorbents. 4(4), 318 10.1016/0167-6989(86)90033-4

[15] Sposito G, Weber JH. Sorption of trace metals by humic materials in soils and natural waters. 1986 5; *Crit*. *Rev*. *Environ*. *Control*. 16(2), 193–229. 10.1080/10643388609381745

[16] Nebbioso A, Piccolo A. Advances in humeomics: Enhanced structural identification of humic molecules after size fractionation of a soil humic acid. 2012 3; *Anal*. *Chim*. *Acta*. 720, 77–90. 10.1016/j.aca.2012.01.027 22365124

[17] Flaig W, Beutelspacher H, Rietz E. Chemical composition and physical properties of humic substances. 1975; *Soil*. *Components*. 1–211. 10.1007/978-3-642-65915-7_1

[18] Woelki G, Salzer R. Thermal investigations of the structure of two humic acid salts by in situ FTIR spectroscopy. 1995 1; *Fresenius*. *J*. *Anal*. *Chem*. 352(5), 529–531. 10.1007/bf00323379

[19] Chen H, Berndtsson R, Ma M. et al Characterization of insolubilized humic acid and its sorption behaviors. 2009 6; *Environ*. *Geol*. 57, 1847–1853. 10.1007/s00254-008-1472-0

[20] Akbari F, Khodadadi M, Panahi A H, Naghizadeh A. Synthesis and characteristics of a novel FeNi_3_/SiO_2_/TiO_2_ magnetic nanocomposites and its application in adsorption of humic acid from simulated wastewater: study of isotherms and kinetics. 2019; *Environ*. *Sci*. *Pollut*. Res.26, 1–12. 10.1016/j.jclepro.2018.01.052 31605358

[21] Yustiawati, Kihara Y, Sazawa K, Kuramitz H, Kurasaki M, Saito T, et al Effects of peat fires on the characteristics of humic acid extracted from peat soil in Central Kalimantan, Indonesia. 2014 10; *Environ*. *Sci*. *Pollut*. *Res*. 22(4), 2384–2395. 10.1007/s11356-014-2929-1 24781330

[22] Fong SS, Mohamed M. Chemical characterization of humic substances occurring in the peats of Sarawak, Malaysia. 2007; Org. *Geochem*. 38(6), 967–976. 10.1016/j.orggeochem.2006.12.010

[23] Schnitzer M, Hoffman I. Thermogravimetry of soil humic compounds. 1965 8; *Geochim*. *Cosmochim*. *Ac*. 29(8), 859–870. 10.1016/0016-7037(65)90083-9

[24] Szot K, Góralczyk K, Michalska M, Veryho N, Chojnowski J, Ponikowska I, et al The effects of humic water on endothelial cells under hyperglycemic conditions: inflammation-associated parameters. 2019 6; *Environ*. *Geochem*. *Hlth*. 10.1007/s10653-018-0238-1 PMC6702181 30610442

[25] Junek R, Morrow R, Schoenherr JI, Schubert R, Kallmeyer R, Phull S, et al Bimodal effect of humic acids on the LPS-induced TNF-α release from differentiated U937 cells. 2009 1; *Phytomedicine*. 16(5), 470–476. 10.1016/j.phymed.2008.10.003 19131228

[26] Khil’ko SL, Semenova RG. Interaction of humic acid salts with drug preparations. 2016 12; *Solid*. *Fuel*. *Chem*. 50(6), 390–394. 10.3103/s0361521916060057

[27] Proidakov AG. Humic acids from mechanically treated coals: A review. 2009 2; Solid. Fuel. Chem. 43(1), 9–14. 10.3103/s0361521909010030

[28] Kautenburger R, Hein C, Sander JM, Beck HP. Influence of metal loading and humic acid functional groups on the complexation behavior of trivalent lanthanides analyzed by CE-ICP-MS. 2014 3; *Anal*. *Chim*. *Acta*. 816, 50–59. 10.1016/j.aca.2014.01.044 24580854

[29] Schafer H N S. Determination of carboxyl groups in low-rank coals. 1984 5 Fuel. 63(5), 723–726. 10.1016/0016-2361(84)90178-9

[30] Swift RS, Thornton BK, Posner AM. Spectral characteristics of a humic acid fractionated with respect to molecular weight using an agar gel. 1970; *Soil*. *Sci*. 110(2), 93–99. 10.1097/00010694-197008000-00003

[31] Tombficz E, Varga K, Sznnto F. An X-ray diffraction study of alkylammonium humate complexes. 1988 8; *Colloid*. *Polym*. *Sci*. 266(8), 734–738. 10.1007/bf01410283

[32] Wilson MA, Collin PJ, Tate KR. ^1^H-nuclear magnetic resonance study of a soil humic acid. 1983 7; *J*. *Soil Sci*. 34(2), 297–304. 10.1111/j.1365-2389.1983.tb01035.x

[33] Malcolm RL. Factors to be considered in the isolation and characterization of aquatic humic substances. 2006 10; *Lecture Notes in Earth Sciences*. 7–36. 10.1007/bfb0010455

[34] Melton JR, Kantzas A, Langford CH. Nuclear magnetic resonance relaxometry as a spectroscopic probe of the coordination sphere of a paramagnetic metal bound to a humic acid mixture. 2007 12; *Anal*. *Chim*. *Acta*. 605(1), 46–52. 10.1016/j.aca.2007.10.017 18022410

[35] Francioso O, Montecchio D, Gioacchini P, Ciavatta C. Thermal analysis (TG–DTA) and isotopic characterization (^13^C–^15^N) of humic acids from different origins. 2005 10; *Appl*. *Geochem*. 20(3), 537–544. 10.1016/j.apgeochem.2004.10.003

[36] Fukushima M, Furubayashi K, Fujisawa N. Characterization of humic acids in sediments from dam reservoirs by pyrolysis-gas chromatography/mass spectrometry using tetramethylammonium hydroxide: Influence of the structural features of humic acids on iron(II) binding capacity. 2011 7; *J*. *Anal*. *Appl*. *Pyrol*. 9(2),323–331. 10.1016/j.jaap.2011.03.008

[37] Thompson SO, Chesters G. Nonaqueous titration of acidic groups of Lignins. Titration in pyridine using potassium methoxide as titrant. 1964 3; *Anal*. *Chem*. 36(3), 655–657. 10.1021/ac60209a027

[38] Butler JP, Czepiel TP. Determination of phenolic groups in lignin preparations titration with potassium methoxide using dimethylformamide as a solvent. 1956 9; *Anal*. *Chem*. 28(9), 1468–1472. 10.1021/ac60117a035

[39] Liu D, Chen HH. Determination of phenolic hydroxyl and carboxyl groups in lignin with the conductometric titration in organic solvent. 1992; *Cellulose Chemistry & Technology*. 26, 63–71.

[40] Burges NA, Hurst HM, Walkden B. The phenolic constituents of humic acid and their relation to the lignin of the plant cover. 1964 10; *Geochim*. *Cosmochim*. *Acta*. 28(10–11), 1547–1554. 10.1016/0016-7037(64)90005-5

[41] Van DLMJHA. Reactions of humic materials with methionine and arginine. 1979 6; *Soil Biol*. *Biochem*, 11(6), 571–576. 10.1016/0038-0717(79)90024-5

[42] García M, Collado S, Oulego P, & Díaz M. The wet oxidation of aqueous humic acids. 2020 2; *J*. *Hazard*. *Mater*, 396, 122402 10.1016/j.jhazmat.2020.122402 32298859

